# Attraction and oviposition preferences of *Phlebotomus papatasi* (Diptera: Psychodidae)*,* vector of Old-World cutaneous leishmaniasis, to larval rearing media

**DOI:** 10.1186/s13071-015-1261-z

**Published:** 2015-12-30

**Authors:** Bahjat Fadi Marayati, Coby Schal, Loganathan Ponnusamy, Charles S. Apperson, Tobin E. Rowland, Gideon Wasserberg

**Affiliations:** Department of Biology, University of North Carolina at Greensboro, 235 Eberhart Bldg., Greensboro, NC 27402 USA; Department of Entomology, North Carolina State University, Raleigh, NC 27695 USA; Entomology Branch, Walter Reed Army Institute of Research, 503 Robert Grant Avenue, Silver Spring, MD 20910-7500 USA

**Keywords:** Leishmaniasis, Oviposition behavior, Sand flies, Semiochemicals, Bioassay, Olfactometer

## Abstract

**Background:**

As part of a project aimed at developing oviposition attractants for the control and surveillance of *Phlebotomus papatasi* (a vector of Old-World cutaneous leishmaniasis), we tested the hypothesis that gravid sand flies are attracted to chemical cues emanating from the growth medium of conspecific larvae - predominantly larvae-conditioned host feces that represents a suitable oviposition site. We report the results of a systematic assessment of media from various developmental stages of the sand fly using oviposition and olfactometer behavioral assays.

**Methods:**

We conducted multiple-choice oviposition assays in 500 mL Nalgene jars. Six treatments were placed on separate filter paper discs at the bottom of the jar: 2^nd^/3^rd^ larval instar medium, 4^th^ larval instar/pupae medium, frass from expired colonies, larval food (aged rabbit chow and rabbit feces mix), rabbit feces, and a solvent (water) control. Fifty gravid females were introduced into each jar. Cumulative number of eggs laid on each filter paper per jar was counted at different time intervals from digital images. Attraction of gravid sand flies to these six treatments was assayed with a 3-chamber linear olfactometer. Twenty gravid females were transferred to the middle chamber of the olfactometer and their distribution in treatment and control chambers was recorded after 3 h.

**Results:**

Almost no eggs were oviposited during the first 72 h following a blood-meal. Cumulative egg deposition increased drastically in the next 24 h (hours 73–96), with a slight non-significant increasing trend thereafter. Comparing mean cumulative egg deposition among the six treatments, we found that significantly more eggs were oviposited on 2^nd^/3^rd^ larval rearing medium followed by 4^th^ instar/pupae rearing medium. Oviposition preference did not vary over time. The olfactometer results were consistent with the oviposition assays, with 2^nd^/3^rd^ larval rearing medium being the most attractive, followed by 4^th^ instar/pupae rearing medium.

**Conclusion:**

The key finding of this study is that gravid, laboratory reared, *Ph. papatasi* sand flies are significantly more attracted to rearing medium of the most biologically active larval stages (2^nd^/3^rd^ instar and 4^th^ instar/pupae). This finding indicates that sand fly-digested host food and feces is attractive to gravid females and suggests that the larvae and larval gut microbiome may be involved in conditioning the oviposition substrate and possibly the production of oviposition attractants and stimulants.

## Background

Phlebotomine sand flies can transmit protozoan parasites (*Leishmania* spp.), as well as bacterial (*Bartonella bacilliformis*) and viral pathogens (e.g., sand fly fever) [1–4]. Most significant are the human leishmaniases that, following malaria and dengue, are the most pervasive vector-borne diseases [5, 6]. Unfortunately, cost, access, and side effects limit the applicability of existing therapeutic treatments. Therefore, given that no vaccine yet exists, reducing exposure to sand fly bites is the most prevalent disease prevention approach [5, 6]. Sand fly control comprises three general approaches: personal protection (e.g., repellents, insecticide-treated clothing or bed-nets), reservoir host control (e.g., rodent removal using rodenticides or burrow plowing), and residual spraying with insecticides [7, 8]. The most common approach is residual spraying of insecticides; however, the effectiveness of this approach is highly variable, non-specific, and can drive the evolution of insecticide resistance [7, 9, 10]. Source reduction using biolarvicides is often used to control some mosquito species, but since sand fly larvae are terrestrial this approach is not practical [11]. Unlike most biting Nematocera, sand flies develop in terrestrial habitats where eggs are typically laid in soil rich in organic material on which the larvae feed and develop through four instars before pupation and adult emergence. The difficulty of finding breeding sites for sand fly control is an important constraint limiting the application of larvicides [12–14]. Hence, a more focused, biologically-based, and targeted control method is urgently needed [8].

An alternative approach to delivery of the insecticide to the vector is to bring the vector to the insecticide using attractants [15]. This attract-and-kill approach is commonly used to control agricultural pests and disease vectors using sex pheromones, host odors, sugar meal sources, and bacterial mediated oviposition site attractants [16–19]. In the context of controlling disease vectors, oviposition-site attractants are expected to be the most effective because they lure physiologically older females that have blood-fed at least once and are, therefore, more likely to be infected with pathogens [18, 20]. Therefore, by targeting gravid females, control efforts can simultaneously reduce pathogen transmission and control population growth [20].

Most research on oviposition attractants of disease vectors has focused on mosquitoes [20, 21]. With sand flies, most research has focused on *Lutzomyia longipalpis* (Diptera: Psychodidae), the main vector of New-World visceral leishmaniasis [3]. In a series of experiments, conspecific eggs were found to enhance oviposition and dodecanoic acid was identified as the active compound from eggs [22]. Organic matter also stimulates oviposition in *Lu. longipalpis* and hexanal and 2-methyl-2-butanol were isolated from fresh chicken or rabbit feces as the active compounds [23–26]. In contrast, only few studies have examined oviposition in Old-World sand flies. *Phlebotomus papatasi*, the main vector of Old-World cutaneous leishmaniasis (due to *Leishmania major*), is distributed from Morocco to the Indian subcontinent and from southern Europe to central and eastern Africa [3, 4, 27]. It was shown to lay more eggs on substrates containing conspecific eggs [28, 29] or organic matter of various sources [29, 30]. For example, Wasserberg and Rowton [28] compared the relative effectiveness of conspecific eggs and organic matter (frass extract) and found frass to be a much more potent oviposition stimulant than eggs; they also found that the combination of eggs and frass was not more effective than frass alone. Schein et al. [30] showed in the field that cow manure is highly attractive to gravid and non-gravid *Ph. papatasi* females. Chelbi et al. [31] demonstrated that rabbit feces was highly attractive to *Ph. papatasi* in peridomestic environments in Tunisia. Wasserberg [32] used fresh rabbit feces as bait and was able to attract *Ph. papatasi* from as far as 250 m from the nearest potential source. Radjame et al. [33] isolated soil bacteria from a variety of putative sand fly breeding sites (human dwellings, termite mounds, cow sheds) and tested their effect on oviposition responses of gravid females. In bioassays of soil bacterial isolates, *Bacillus licheniformis* and *Staphylococcus saprophyticus* were shown to enhance *Ph. papatasi* oviposition response.

Our general goal is to discover, develop and optimize a lure that attracts oviposition-site seeking gravid females and that could be used for surveillance and control of *Ph. papatasi* sand flies. Because larval sand flies are coprophagic [1, 3, 12], we hypothesized that gravid sand flies are attracted to chemical cues associated with the decomposition of organic matter of (predominantly) fecal origin as indicators of suitable oviposition sites. Specifically, given our previous observation that larval rearing substrate is substantially more effective than conspecific eggs in inducing egg deposition [28], our goal in this study was to compare the attraction and oviposition response of gravid *Ph. papatasi* females among rearing substrates of pre-larval, larval, and post-larval stages in order to identify the most attractive and oviposition-stimulating source material. In this paper, we report the results of the screening of these potential attractant sources using oviposition and olfactometer behavioral assays.

## Methods

### Insects and colony maintenance

*Phlebotomus papatasi* sand flies originating from Abkük, Turkey (37.39103°N 27.43853°E), were colonized at the Walter Reed Army Institute of Research (Silver Spring, Maryland) and maintained at the University of North Carolina in Greensboro. Rearing of *Ph. papatasi* sand flies followed the mass-rearing methods described by Modi and Rowton [34] and flies were blood-fed on live anesthetized ICR mice (Harlan) (UNCG IACUC protocol 14–07). Sand flies were maintained in incubators (Model: 6030–1, Caron®, Marietta, Ohio) at 26 °C, 80 % RH, and 12:12 light:dark cycle. Colonies were maintained in 500 mL Nalgene jars (Nalgene™, Model 81063, diameter = 11 cm) with a 2.2 cm layer of Whip-Mix® Orthodontic Plaster (Model: 5577352, Henry Schein Inc., Melville, New York) on the bottom to ensure moist substrate and drainage. Larval food was prepared by mixing fresh rabbit feces (New Zealand White strain) and rabbit chow (Purina) at a 1:1 ratio, which was fermented for 3 weeks in a dark chamber, air-dried and ground to a powder.

### Treatments for oviposition and olfactometer assays

Source material included rearing substrate of two pre-larval stages, two larval stages, and one post-larval stage. Pre-larval stage substrates included fresh ground rabbit feces (RF) and unused larval food (LF) (see description above). Larval stage substrates included substrate containing mainly larvae at the 2^nd^ and 3^rd^ instar (2^nd^/3^rd^ substrate) or 4^th^ instar and pupal stages (4^th^/pupae substrate). Post-larval substrate was rearing medium of a colony jar from which all sand fly pupae had eclosed (hereafter “expired”).

### Oviposition assays

**Fig. 1:**
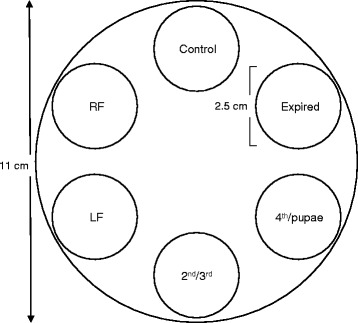
Six-choice oviposition assays. Each assay jar was constructed of a 500 mL cup with 2.5 cm diameter filter paper discs distributed at equal distance. Six source materials were placed on the filter papers: Control (water-only); rabbit feces RF; larval food (LF); rearing medium from 2^nd^ and 3^rd^ larval instars (2^nd^/3^rd^); rearing medium from 4^th^ larval instars and pupae (4^th^/pupae); rearing medium and frass from an expired colony (Expired)

We conducted multiple-choice behavioral assays using 500 mL Nalgene jars (similar to the rearing jars) modified for 6-choice assays. Each jar was placed in water for 12 h prior to the start of an experiment to equilibrate the moisture level of its plaster floor. We simultaneously tested the above described five source materials and a solvent (water-only) negative control treatment (Fig. 1). To minimize the potential of cross-contamination, 1 mg of each of these materials (SE = 0.1 mg) was placed on a filter paper disc (2.5 cm diameter) (Model: 09-801-AA, ThermoFisher Scientific®, Waltham, Massachusetts) at equal distance from the center of the cup. Three drops (~0.15 mL) of deionized water were then added to each filter paper. Each experimental session (*n* = 9 replicate sessions conducted between 3/1/2013 and 4/17/2013) consisted of 7 oviposition jars. During the first 24 h post blood-meal sand flies were left undisturbed in their holding cage to not interrupt the development of the peritrophic matrix around their recently acquired blood-meal [34]. Then, fifty gravid females were transferred into each of the 7 bioassay jars using a mouth aspirator. Jars were then returned into the rearing incubator. To obtain a time-course of oviposition, the assays were terminated, one jar at a time, 1–7 days after transfer (or 2–8 days post blood-meal) by releasing the females into a separate holding cage. We photographed the filter-papers with a T3i Canon 100 mm macro lens. Eggs laid on each filter paper were counted from high quality digital photos using the counting tools in Adobe Photoshop (Adobe Photoshop CS5 2010, Adobe™, San Jose, California).

### Attraction bioassays

**Fig. 2:**
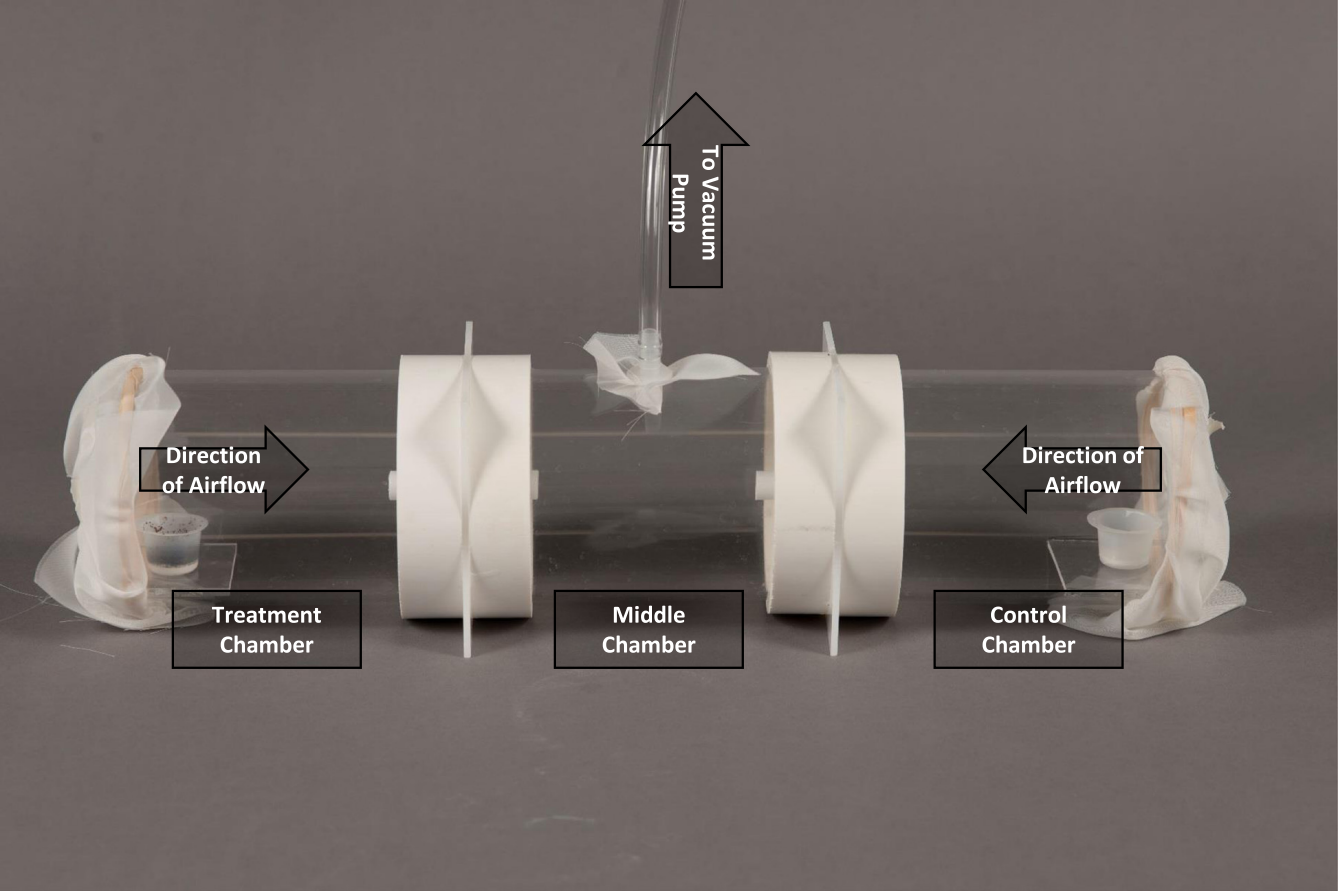
Three-chamber in-line olfactometer. The olfactometer was constructed so that the vacuum pump drew air across the treatment and control cups and into the middle chamber, where 20 gravid females were introduced. Weigh boats containing test or control materials were placed on a small shelf at the end of each side chambers. Chambers were connected by 6 cm long (1 cm inner diameter) tubes extending 3 cm into both the side chamber and the central chamber

Attraction of gravid sand flies to various source materials was assayed with a 2-choice olfactometer (Fig. 2). Briefly, the olfactometer consisted of a cylindrical Plexiglas® apparatus made of three in-line chambers (each chamber: 9.4 cm inner diameter, 10.1 cm outer diameter, 15 cm length). A section of polyvinyl chloride (PVC) pipe (2.5 cm length, 10.15 cm inner diameter), glued to either side of a white Plexiglas square partition (11.4 × 11. 4 cm, 3 mm thickness), coupled the middle chamber to the outer two chambers. Holes in the center of each partition held a 6 cm long (1 cm inner diameter) tube extending 3 cm into the central chamber and 3 cm in an outer chamber. In each olfactometer, test material was placed in one side chamber and the control material in the other side-chamber. Test material (0.5 g) to be tested was placed on a 7.5 mL weigh boat containing 1.2 mL of orthodontic plaster and tested against a blank negative control (similar plaster-bottomed weigh boats but with 3 water drops [ca. 0.15 mL]). In each experimental session (*n* = 10 replicate sessions conducted between 12/4/2013 and 2/2/2014), we used six olfactometers with source materials including: 2^nd^/3^rd^ substrate, 4^th^/pupae substrate, “expired’ colony substrate, LF, and RF as well as one olfactometer with blank (water) controls on both sides to test for potential directionality bias. A treatment weigh boat was placed on a plastic stage at one end of the olfactometer, and the other end received a control weigh boat. The ends of the side-chambers were then covered with a fine mesh screen secured with rubber bands (Fig. 2). Twenty gravid *Ph. papatasi* females (72 h post blood-meal) were transferred to the middle chamber of the olfactometer. The middle chamber was then connected to a vacuum pump (Air Admiral® Cole-Parmer, Vernon Hills, IL) that delivered a total volumetric flow of 1.05 L/min (~7.5 cm/s through each outer chamber). The vacuum pump remained off for the first 60 min of the bioassay and then on for 2 h. The olfactometer was then placed into a −20 °C freezer to kill the flies and subsequently the number of females in each chamber was counted. Before each bioassay, olfactometers were cleaned using an odorless cleaning detergent (RBS-35, Model: 27950, ThermoFisher Scientific, Waltham, Massachusetts). All bioassays were conducted in a controlled environment room with temperature and humidity identical to those of the rearing colony incubator. Assays were conducted in the scotophase 3–8 h after lights-off. The olfactometers were randomly assigned locations within the room to avoid directional bias. Treatment side was rotated among replicate session.

### Statistical analysis

#### Oviposition assays

In these experiments, data represented cumulative number of eggs laid over a specified number of days until experimental termination of oviposition. To analyze the oviposition time-course, we used the cumulative number of eggs per female per jar. Data were analyzed using Kruskal-Wallis test. To compare the cumulative egg number between the six source materials within each jar (treatments clustered within jars) and to account for the nature of the data (overdispersed, count data), we used random-intercept negative-binomial multiple regression [34]. Specifically, we tested for the effect of source material (as dummy variables), time since blood-meal, and their interaction, on the cumulative number of eggs laid per filter paper disc.

#### Attraction assays

An Oviposition Attraction Index (OAI) [35] was used to evaluate and compare the responses of gravid sand flies to source materials of different types. This index was calculated as OAI = (N_t_–N_c_)/(N_t_ + N_c_) where N_t_ and N_c_ are the number of females found in the test or control chambers of the olfactometer, respectively. We used linear regression to test the effect of the different source materials (treated as dummy variables) on OAI. Since OAI statistical distribution is truncated between −1 to +1, we used a robust estimate of the standard error that accounts and corrects for possible violations of normality [34]. For all analyses, significance level of *P* < 0.05 was used. Analysis was conducted using Stata software (StataCorp., College Station, TX).

## Results

### Oviposition preferences in multiple-choice assays

#### Time-course of oviposition

**Fig. 3:**
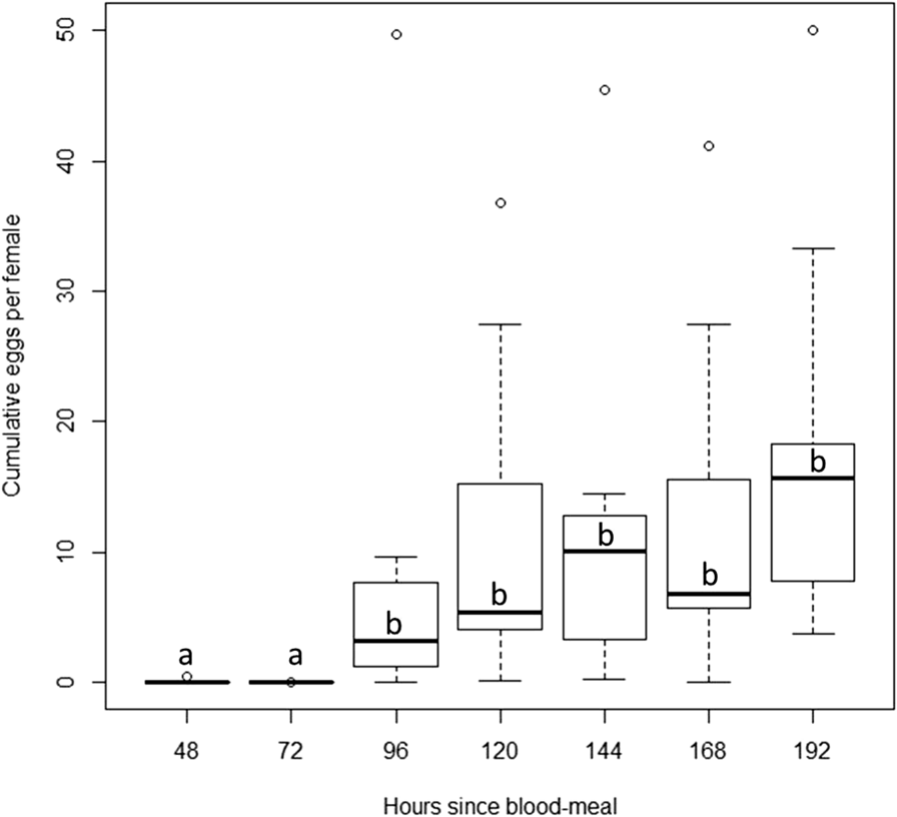
Boxplot describing time course of egg deposition: cumulative number of eggs/female/jar over time since blood-meal. Black bars indicate median and box represent the second-to-third inter-quartile range. Letters indicate significant difference in egg numbers among time periods

Almost no eggs were oviposited during the first 72 h following the blood-meal. Subsequently, cumulative egg deposition increased significantly (Kruskal-Wallis: *χ*^2^ = 31.08, df = 1, *P* < 0.0001) with a slight non-significant increasing trend thereafter (Kruskal-Wallis: χ ^2^ = 5.842, df = 4, *P* = 0.21) (Fig. 3). Mean per-capita egg deposition for this period (days 4 to 8 post blood-meal) was 13.25 (SE = 2.10).

#### Preferences of oviposition substrate

**Fig. 4:**
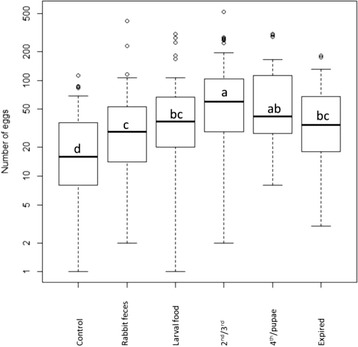
Boxplot describing the effect of various rearing media on the cumulative number of eggs oviposited per filter paper disc (drawn on a log10-scale) in 6-choice oviposition jars. Black bars indicate median and box represent the second-to-third inter-quartile range. Letters indicate significant difference in egg numbers among substrate type

Since almost no eggs were oviposited during the first 72 h following the blood-meal, statistical analysis was performed for data of the subsequent days (days 4 to 8 following blood-meal). Significantly more eggs were oviposited on each of the tested substrates than on the water-only control (Table 1)*.* The highest number of eggs was oviposited on 2^nd^/3^rd^ larval rearing medium followed by 4^th^/pupae rearing medium (Table 1, Fig. 4). There was no significant difference between these top two preferred substrates, but 2^nd^/3^rd^ larval rearing medium had significantly more eggs than the three lower ranking substrates (Table 1, Fig. 4). There were no significant differences among the three lower ranking substrates (Table 1). As indicated by a non-significant treatment-by-time interaction term (random intercept model: Z = 1.61, *P* = 0.11) the relative preference for the different substrates did not change over time.

**Table 1:** Oviposition preferences in multiple-choice assays. Random-intercept negative-binomial regression table of the effect of different oviposition substrates on the cumulative number of eggs oviposited per filter paper disc in 6-choice oviposition assays. Table also presents means (±SE) of egg numbers oviposited per filter paper disc for each substrate type. Test materials included larval rearing media of different types and stages including: fresh rabbit feces (RF), fresh larval food (LF), rearing medium containing frass of 2^nd^-3^rd^ instar larvae (2^nd^/3^rd^), rearing medium containing frass of 4^th^ instar larvae and pupae (4^th^/pupa), frass of rearing cups from which all larvae had eclosed (expired) and a negative (water) control. Rearing media of 2^nd^/3^rd^ and 4^th^/pupa (bolded) induced highest oviposition response

Substrate	Mean number of eggs (SE)	Regression Coefficient	SE	Z	P
Control	25.91 (4.69)	0.105	0.155	0.67	0.501
RF	49.86 (12.42)	0.464	0.173	2.68	0.007
LF	63.43 (12.45)	0.671	0.169	3.96	<0.0001
**2** ^**nd**^ **/3** ^**rd**^	**95.73 (18.13)**	**1.006**	**0.162**	**6.19**	**<0.0001**
**4** ^**th**^ **/pupae**	**72.51 (12.91)**	**0.899**	**0.163**	**5.51**	**<0.0001**
Expired	48.08 (7.20)	0.607	0.169	3.58	<0.0001
Ln r		0.648	0.244		
Ln s		3.38	0.32		
r		1.91	0.47		
s		29.42	9.31		

r, s: Negative binomial dispersion parameters

Likelihood-ratio test for overdispersion: *χ* ^2^ = 109.28, *P* < 0.0001

### Olfactometer attraction assays

We tested the attractiveness of the five substrates in olfactometer assays. Only data from bioassays in which ≥25 % of the females responded were included. No significant bias was found for olfactometers with water-only controls on both sides, as a mean of 4.33 (SE = 0.31) flies chose the right-side chamber and 4.25 (SE = 0.51) flies were in the left-side chamber of the olfactometer (paired *t* = 0.134, *P* = 0.895) (Table 2).

**Table 2:** Olfactometer attraction assays. Mean numbers (±SE) of *Ph. papatasi* females in the treatment, middle, and control chambers of the olfactometers and percent flies responding (Total number of flies at both the treatment and control chambers divided by total flies used) for the different substrate types. Rearing media of 2^nd^/3^rd^ and 4^th^/pupa (bolded) induced highest attraction

Substrate	No. flies	Treatment	Middle	Control	OAI	% response	P
^a^Control	20	4.33 (0.31)	11.42 (0.57)	4.25 (0.51)	0.04 (0.07)	43 (2.8)	0.600
RF	20	4.9 (0.18)	12.4 (0.34)	2.7 (0.3)	0.30 (0.06)	38 (17)	0.002
LF	20	6.4 (0.54)	9.5 (0.73)	4.2 (0.29)	0.20 (0.03)	53 (3.8)	0.028
**2** ^**nd**^ **/3** ^**rd**^	**20**	**7.2 (0.63)**	**10.9 (0.57)**	**1.9 (0.23)**	**0.56 (0.06)**	**46 (2.8)**	<**0.0001**
**4** ^**th**^ **/pupae**	**20**	**6.2 (0.61)**	**11.3 (0.7)**	**2.5 (0.17)**	**0.41 (0.04)**	**44 (3.3)**	<**0.0001**
Expired	20	4.6 (0.31)	11.9 (0.43)	3.4 (0.34)	0.18 (0.04)	40 (2.1)	0.084

^a^The right chamber of the olfactometer was assigned as “treatment” and left chamber as “control”, but both received water-only

**Fig. 5:**
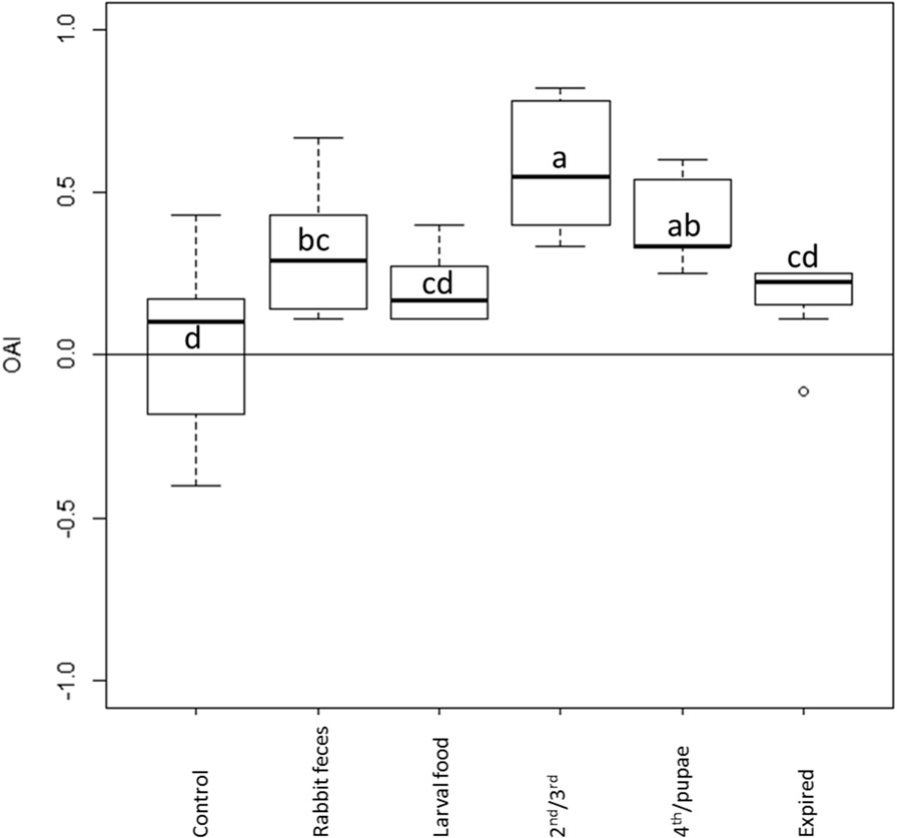
Boxplot describing the effect of rearing media on the oviposition attraction index (OAI) of *Ph. papatasi* sand flies as measured in olfactometer assays. Black bars indicate median and box represent the second-to-third inter-quartile range. Letters indicate significant difference in OAI among substrate type

Overall, 44 % of the flies responded (i.e., moved to the two sides of the chambers), while 66 % remained in the central chamber; there were no significant differences among treatments, but the ‘larval food’ treatment elicited a higher total response than the other treatments (Z = 2.13, *P* = 0.033) (Table 2). Sand fly females were significantly attracted to four of the five tested materials. As in the 6-choice experiment, 2^nd^/3^rd^ larval rearing substrate was the most attractive material, followed by 4^th^/pupae rearing substrate (Fig. 5, Table 2). ‘Rabbit feces’ was the next most attractive substrate. However, ‘larval food’ and ‘expired colony’ substrates did not significantly differ from the control (Fig. 5). Furthermore, the effect of the expired colony substrate was not statistically (albeit marginally) significant (Table 2).

## Discussion

The key finding of this study is the observation that gravid *Ph. papatasi* females were attracted to and stimulated to oviposit in rearing medium of the most biologically active larval stages (2^nd^/3^rd^ and 4^th^/pupae). Our experiments clearly indicate that untreated rabbit feces were less attractive and stimulated fewer oviposition events than 2^nd^/3^rd^ larval substrate. Furthermore, adding rabbit chow and fermenting this mix for 3 weeks (larval food preparation process) also did not enhance attraction or oviposition. Only when larval substrate was conditioned through ingestion by foraging larvae were both attraction and oviposition enhanced. This ingestion-mediated conditioning suggests the involvement of digestive processes and the gut microbiome in enhancing the attractiveness of this substrate [36]. Gut microbes are not known to be vertically transmitted in sand flies [37], and the source of their gut microbial community is the environment [36, 38, 39]. In our experiments, gut microbes likely originated from rabbit feces and larval food. Nonetheless, the larval gut can shape the microbial community as it facilitates the proliferation of some microbes and inhibits others. The idea that bacteria contribute to attraction of gravid sand flies is further supported by our preliminary analysis showing that a mixture of bacterial isolates from this substrate is as attractive to females as the solid substrate (Kakumanu et al. unpublished data). Furthermore, some of the most attractive bacterial isolates belong to taxa that include insect gut bacteria. Our ongoing research aims to determine the relative contributions of substrate aging and its conditioning by larvae to attractiveness of the substrate to gravid sand flies.

We do not yet understand the underlying evolutionary reasons for the patterns of oviposition site selection observed here. However, in most species with relatively sedentary larvae, females tend to seek oviposition sites that maximize larval survival, most often host plants or suitable food resources. Given that decomposing organic matter is the main food source for sand fly larvae [1, 3] we hypothesized that natural selection has molded oviposition site-seeking females to detect and orient to olfactory cues that signal the availability of food for their larvae. Indeed, almost all organic matter media that we tested were more attractive and stimulated females to oviposit more than the water control. But not all organic substrates were equally attractive. Larval substrate became more attractive as larvae matured, but then its attractiveness gradually declined as larvae further matured, pupated and eclosed. This initial increase in attraction might appear maladaptive, as older sand fly larvae might be cannibalistic [40]. Yet, as suggested by Wasserberg et al. [21] with respect to mosquitoes, the intraspecific regulation of oviposition site selection is a complex process involving trade-offs between attraction at low-to-medium conspecific densities, where presence of conspecifics indicates site suitability, and repellence/deterrence at high densities that indicate potential adverse competitive effects. This results in a hump-shaped (upside down parabola) curve describing the relationship between attraction and conspecific densities. It is possible that a similar process occurs here in relation to ecological succession of microbes in the rearing medium, with 2^nd^/3^rd^ stage substrate occurring at the optimum successional time-point.

The time-course of oviposition following a blood-meal indicated that *Ph. papatasi* sand flies did not start laying eggs within 72 h after a blood-meal (Fig. 3). Subsequently, oviposition sharply increased followed with a slight increasing trend until day 8. Thus, sand flies laid most of their egg clutch once they became physiologically capable of doing so (72–96 h post blood-meal) and then laid additional 1.8 eggs (per capita) approximately every 24 h. Schlein et al. [41] did not observe a sudden increase in oviposition at a particular day but they did observe continuously increasing cumulative egg number between days 7 and 14 post blood-meal. Per-capita egg deposition observed in our study (13.25 per female) is lower than the 15–20 eggs-per-female previously reported by Wasserberg and Rowton [28] or the 33.44 eggs-per-female observed by T. Rowland (personal observations) for individually-reared *Ph. papatasi*. Given that this experiment took place between early January to mid-April, these lower egg numbers might possibly be related to photoperiodic fluctuation in oviposition activity as previously observed by Schlein et al. [41] who found substantially lower egg-deposition levels during the late fall to early winter period compared with those observed during *Ph. papatasi*’s typical activity period (May – October). It is also interesting to note that time-to-oviposition as observed here is shorter than that observed by Volf and colleagues [40, 42] who report 7 days to first oviposition under similar rearing conditions.

We used the oviposition time-course results to guide our olfactometer experiments where we used only females 72–96 h post blood-meal to ensure females were gravid and at the stage where they would be seeking a suitable oviposition site and therefore should be responsive to olfactory cues. In addition, we observed that oviposition-substrate preference of gravid *Ph. papatasi* females did not change significantly over time. This finding is in contrast to Elnaiem et al. [24] who noted that oviposition preference switched from rabbit feces to the water control for *Lu. longipalpis* between days 3–4 and day 5 post blood-feeding. Nevertheless, our experimental design using cumulative oviposition might not be ideally suited for detecting temporal changes in oviposition-substrate preference and day-specific bioassays for a fixed oviposition time window might be better suited.

In conclusion, the sensory acuity of female sand flies to distinguish between rearing media of different larval developmental stages which are apparently very similar is quite remarkable. Our results suggest the involvement of larval gut microbial community in the production of oviposition attractants. Indeed, Peterkova-Koci et al. [39] showed that *Lu. longipalpis* prefers laying eggs on rabbit feces containing its original bacterial assemblage compared with sterile feces. Furthermore, they showed that these bacteria are beneficial to the sand fly in terms of larval growth and survival. Yet, de-coupling the effect of substrate aging by itself from its conditioning by feeding larvae still warrants further study. The chemical- and microbial-ecology processes driving this behavior are still not understood and are currently being investigated by our group. Finally, once optimal attractive blends are formulated, we will test them in the field.

## Conclusion

We found that rearing medium of 2^nd^/3^rd^ instar *Ph. papatasi* is substantially more attractive than the pre-larval (rabbit feces, fresh larval food) or post-larval (expired colony medium) rearing media. These results suggest that larval digestion and possibly the larval gut microbial community contribute to the production of oviposition attractants. Identifying these microbes and the attractive compounds they produce would lead the way for the development of an attractive lure to be used for the surveillance and control of sand flies.
